# Family well-being as an important protective factor in the treatment of stress and emotional burnout among medical professionals during the COVID-19 pandemic in Russia

**DOI:** 10.1192/j.eurpsy.2021.829

**Published:** 2021-08-13

**Authors:** J. Koniukhovskaia, E. Pervichko, O. Mitina, O. Stepanova, E. Dorokhov

**Affiliations:** 1 Psychology, Lomonosov Moscow State University, Moscow, Russian Federation; 2 Faculty Of Psychology, Lomonosov Moscow State University, Moscow, Russian Federation; 3 Faculty Of Psychology And Social Sciences, Pirogov Russian National Research Medical University, Moscow, Russian Federation

**Keywords:** stress, emotional burnout, medical professionals, COVID-19 pandemic

## Abstract

**Introduction:**

The COVID-19 pandemic and the need to fight it disrupt the balance between work and rest for health workers that can lead to a decrease in stress tolerance and emotional burnout appearance. The lifestyle and well-being of personal and family life can be both a “depletion” and a “resource” factor for health professionals when working under stressful conditions.

**Objectives:**

To study the presence/absence and severity of burnout symptoms in medical professionals in the COVID-19 pandemic context; to investigate the interaction between burnout severity and overall stress levels, family well-being, and the presence of children.

**Methods:**

The author’s socio-demographic questionnaire, Stress Perception Questionnaire (Linville, 1987), modified Pandemic Perception Questionnaire (Broadbent et al.,2006), Maslach Burnout Inventory (Maslach et al.^1996), State-Trait Anxiety Inventory (Spielberger et al., 1983) were used. The study was conducted online from April 27 to October 26. It involved 249 medical workers, including 58 men and 191 women.

**Results:**

Health workers who have children show greater confidence in their professional competence(41.28±6.3vs39±7;p=0.007) and (at the trend level) have a lower level of exhaustion(34.53 ±9.2vs36.71±10.8;p=0.09) than their colleagues without children. Although health workers in both groups have approximately the same scores for perceived stress, however, those with children put less effort to counteract stress(9.31±2.5vs10.19±2.9;p=0.012). They describe the pandemic as less dangerous compared to colleagues who do not have children(15.4±5.7vs16.7±5.1;p=0.042).

**Conclusions:**

Perhaps the very possibility of switching attention from a vitally dangerous topic to more positive aspects of life allows medical staff who have children to feel less exhausted and maintain faith in their own strength.

